# Asbestos Fibers Enhance the TMEM16A Channel Activity in Xenopus Oocytes

**DOI:** 10.3390/membranes13020180

**Published:** 2023-02-01

**Authors:** Annalisa Bernareggi, Martina Zangari, Andrew Constanti, Paola Zacchi, Violetta Borelli, Alessandro Mangogna, Paola Lorenzon, Giuliano Zabucchi

**Affiliations:** 1 Department of Life Sciences, University of Trieste, Via Fleming 22, 34127 Trieste, Italy; 2 Department of Pharmacology, UCL School of Pharmacy, 29/39 Brunswick Square, London WC1N 1AX, UK; 3 Department of Life Sciences, University of Trieste, Via Valerio 28/1, 34127 Trieste, Italy; 4 Institute for Maternal and Child Health—IRCCS Burlo Garofolo, Via Dell’Istria 65/1, 34137 Trieste, Italy

**Keywords:** TMEM16A channels, *Xenopus* oocytes, asbestos fibers, crocidolite, voltage clamp

## Abstract

Background: The interaction of asbestos fibers with target cell membranes is still poorly investigated. Here, we detected and characterized an enhancement of chloride conductance in *Xenopus* oocyte cell membranes induced by exposure to crocidolite (Croc) asbestos fibers. Methods: A two-microelectrode voltage clamp technique was used to test the effect of Croc fiber suspensions on outward chloride currents evoked by step membrane depolarization. Calcium imaging experiments were also performed to investigate the variation of ‘resting’ oocyte [Ca^2+^]_i_ following asbestos exposure. Results: The increase in chloride current after asbestos treatment, was sensitive to [Ca^2+^]_e_, and to specific blockers of TMEM16A Ca^2+^-activated chloride channels, MONNA and Ani9. Furthermore, asbestos treatment elevated the ‘resting’ [Ca^2+^]_i_ likelihood by increasing the cell membrane permeability to Ca^2^ in favor of a tonic activation of TMEME16A channels_._ Western blot analysis confirmed that TMEME16A protein was endogenously present in the oocyte cell membrane and absorbed by Croc. Conclusion: the TMEM16A channels endogenously expressed by *Xenopus* oocytes are targets for asbestos fibers and represent a powerful tool for asbestos–membrane interaction studies. Interestingly, TMEM16A channels are highly expressed in many types of tumors, including some asbestos-related cancers, suggesting them, for the first time, as a possible early target of crocidolite-mediated tumorigenic effects on target cell membranes.

## 1. Introduction

Asbestos is a very dangerous fibrous silicate mineral whose inhalation can lead to chronic lung inflammation and aggressive lung and pleural tumors [1]. The fibers can enter the target cell cytosol and reach the nuclear compartment to interfere with DNA integrity and transcriptional activity [2]. Presently, the mechanism of interaction between asbestos fibers and biological cell membranes is still largely unknown. For this reason, we recently successfully proposed *Xenopus* oocytes as a suitable model for studying in detail, the interaction between asbestos fibers and biological cell membranes at the electrophysiological and morphological level [3,4]. We found that following asbestos (Croc, crocidolite) fiber exposure, the biophysical properties of the *Xenopus* oocyte membrane changed: the resting membrane potential became depolarized, the membrane resistance decreased, and more surprisingly, the amplitude of outward currents evoked by step depolarization under voltage clamp increased. We hypothesized that among the possible mechanisms that could be responsible for these effects was the modulation of a Ca^2+^-activated chloride channel (CaCC) [5], most likely TMEM16A, endogenously expressed by the oocytes [6]. This idea was supported by the observations that both asbestos-induced *Xenopus* oocyte cell membrane modifications and TMEM16A activation can be induced by ROS [4,7] and are dependent on the cell actin-cytoskeleton [4,8]. The electrophysiological properties of TMEM16A channels involve intracellular Ca^2+^ and voltage-dependence [9], rectifying properties [10] and ion selectivity [11]. Moreover, their pharmacology is very similar to that of the classical CaCC channels [12]. 

After its identification as a member of the CaCC family, it became clear that the TMEM16A protein is identical to DOG1, a reliable biomarker in gastrointestinal stromal tumours and head and neck cancers [13,14,15]. The overexpression of the TMEM16A is associated with many types of cancer, including gastrointestinal stromal tumors, gastric cancer, head and neck squamous cell carcinoma, colon cancer, pancreatic ductal adenocarcinoma, lung tumours and esophageal cancer [9,13,14,15,16,17]. Here, we tested the hypothesis that TMEM16A channels could mediate the early effects of Croc fiber exposure on *Xenopus* oocyte cell membranes, and we investigated such a possibility, by analyzing whether or not the Croc-mediated effects are influenced by TMEM16A channel blockade using specific TMEM16A channel antagonists such as MONNA [18] and Ani9 [19]. It is now known that an intimate association exists between TMEM16A upregulation and different types of cancer [17] and the fact that asbestos fibers are a well-known trigger of tumorigenesis, suggests that this membrane protein may be a relevant asbestos target capable of initiating/supporting fiber-induced tumor pathogenesis.

## 2. Materials and Methods

### 2.1. Asbestos Fiber Suspensions

Analytical Standard UICC samples of Croc (Croc, South African CAS:12001–28–402704-AB) and chrysotile (Chry “A“ Rhodesian CAS #: 12001-29-5) were obtained from SPI-CHEM, West Chester, Pennsylvania, re-suspended in phosphate-buffered saline (PBS) at a final concentration of 10 mg/mL, and stored at 4 °C until use. Wollastonite (Woll), used as a control particulate, being a non-asbestos silicate powder, was a kind gift of Bal-Co SpA (Sassuolo, MO, Italy). The fiber size parameters of the asbestos UICC standards have been described in detail by Kohyama et al. (1996) [20]. Woll characterization in length distribution was reported by Governa et al. (1998) [21]. Dry asbestos was handled in a Multihazard Glovebox (Vesta I, Bioair, Milan, Italy) to prevent inhalation of the fibers. At the concentration, continuous mixing and temperature used in our experiments, no fiber aggregation occurred as judged by optical microscope analysis. All fiber types in PBS were left to sediment for 2 min to avoid larger fiber aggregates. 

### 2.2. Xenopus Oocyte Preparation

Animal care treatment was conducted in conformity with institutional guidelines in compliance with national (Italian Ministry of Health, authorization number 719/2021-PR) and international laws and policies (European Economic Community, Council Directive 63/2010 Italian D.L. 26/2014). The study is reported in accordance with the available guidelines. The study was approved by the *Organismo Preposto al Benessere degli Animali* (OPBA) of the University of Trieste, and approved by the Italian Ministry of Health, with the authorization number 719/2021-PR. Adult female *Xenopus laevis* frogs were fully anesthetized in cold 0.17% MS-222 solution and oocytes were isolated, as described in detail in Bernareggi et al. (2015) [3].

### 2.3. Electrophysiological Recordings

Electrophysiological recordings were performed 24 h after the isolation to allow healing of the oocyte membrane from damage caused by the collagenase. Ten-fifteen oocytes (stage VI) in a 1.5-mL Eppendorf tube were incubated in 1 ml of Ringer’s solution (NaCl 115 mM, KCl 2 mM, CaCl_2_ 1.8 mM, HEPES 5 mM, adjusted to pH 7.4 with NaOH) without (Ctrl cells) or in test conditions (Croc), in both cases, under continuous mixing for 5–30 minutes (wheel, 7 revolutions/min). Based on the dose–response effect reported in our previous study [3], all experiments were performed at 15 μg/mL of Croc fibers. During the recordings, the cells were continuously superfused with a normal Ringer’s solution or with a Ringer’s solution containing high Ca^2+^ (Ca11: NaCl 95.6 mM, KCl 2 mM, CaCl_2_ 11 mM, HEPES 5 mM, pH 7.4). The measurement of membrane resistance was obtained from the 1/slope of the linear I-V relationship at voltages from −70 to −40 mV; within this range, the currents were flat without rectification. MONNA and Ani9 were purchased from SIGMA. To reduce the variability of oocytes coming from different frog donors, the results were usually compared among oocytes of the same batch. All evoked currents were measured at the peak. Data acquisition and analyses were performed by WinWCP version 4.1.2 Strathclyde Electrophysiology software, kindly provided by Dr John Dempster (Glasgow, United Kingdom). 

### 2.4. Calcium Imaging

For intracellular Ca^2+^ concentration ([Ca^2+^]_i_) measurements, oocytes (stage VI) were injected with Fura 2 free acid (from SIGMA, 36 mL, 1 mM, dissolved in sterilized water) 15 min before the fluorescence image acquisition. Image acquisitions were carried out at RT and, according to the experimental protocol, in Ringer’s solution (as above), in Ringer’s solution containing high Ca^2+^ (Ca11; as above) or in a Ca^2+^-free solution (Ca0; NaCl 95.6 mM, KCl 2 mM, MgCl_2_ 5 mM, EGTA 0.5 mM, HEPES 5 mM, pH 7.4). Oocytes were excited alternately at 340 and 380 nm, selected by a monochromator (PolychromeII, TillPhotonics GmbH, Martinsried, Germany). Fluorescence images were collected by a CCD camera (SensiCam; PCO Computer Optics, Kelheim, Germany) at the acquisition rate of 1 ratio image/s. The monochromator and CCD camera were controlled by TILLVision software (TillPhotonics), also used for image processing. The [Ca^2+^]_i_ levels were calculated offline from the ratio images (340/380) of tested oocytes as mean values of the fluorescence intensity in cell regions of interest. For each experimental group, the mean fluorescence value was then expressed relative to the mean fluorescence value measured in appropriate control oocytes from the same frog donor and expressed as %.

### 2.5. Isolation of a Membrane—Rich Fraction from Xenopus Oocytes

Isolation of *Xenopus* oocyte cell membranes was performed by modification of the method of Clémençon et al. (2014) [22]: briefly, 750 oocytes, stored at −20 °C, were re-suspended in 7 mL of Tris-HCl buffer 20 mM pH 7.0 containing proteinase inhibitors (cOmplete Protease Inhibitor Cocktail, one tablet dissolved in 50 ml extract), homogenized by 60 strokes in a Potter-Elvehjem glass homogeniser equipped with a Teflon motor-operated pestle. The mixture was centrifuged at 1000× *g* for 20 min at 4 °C. Four bands were obtained, fat, cytosol + membrane, black granules and nuclei. The cytosol+membrane fraction was carefully withdrawn and centrifuged in a himac CS150NX micro ultracentrifuge (Hitachi, Tokyo, Japan) for 60 min at 4 °C at 100,000× *g*. The pellet (p100), a membrane rich fraction and the cytosol (s100) were carefully separated. p100 was extracted in 10 mL of 20 mM Tris-HCl pH 7.0 containing 1.5% β−octyl-glucopiranoside (OG) and 1 M NaCl, overnight at 4 °C. The extract was centrifuged at 1000× *g* to avoid precipitates and extensively dialysed against 20 mM Tri-HCl pH 7.0. The dialysate was re-centrifuged at 20,000× *g* in a Hitachi centrifuge to eliminate a few small precipitates of unknown origin and filtered through a 0.2 µm pore size membrane (Minisart filter, Millipore, Merck, Darmstadt, Germany).

### 2.6. Isolation of a Membrane—Rich Fraction from the MeT5a Cell Line

A total of 100 × 10^6^ Met5a cells (a human mesothelial cell line, ATCC CRL-9444) were grown in M199 medium supplemented with 10% heat-inactivated fetal bovine serum (FBS), L-glutamine 2 mM, 100 U mL^−1^ penicillin and 100 U mL^−1^ streptomycin, 3.3 nM epidermal growth factor (EGF), 400 nM hydrocortisone, 870 nM insulin at 37 °C in a 5% CO_2_ atmosphere in 250 ml flasks. Confluent MeT5A cultures were detached with trypsin, washed, counted in a Beckman Coulter Counter Z1, and stored at −20 °C until re-suspended in 25 mL of Tris-HCl buffer 20 mM pH 7.0 containing proteinase inhibitors (cOmplete Protease Inhibitor Cocktail, one tablet dissolved in 50 ml extract). The cells were disrupted in an N_2_-cavitator at 350 psi for 20 min at 4 °C and the lysate centrifuged at 1000× *g* for 20 min at 4 °C. The post-nuclear (PNS) fraction was carefully withdrawn and centrifuged in a himac CS150NX (Hitachi) micro ultracentrifuge for 60 min at 4 °C at 100,000× *g*. The pellet (p100 MET), a membrane rich fraction, and the cytosol (s100) were carefully separated. P100 MET was extracted in 10 mL of 20 mM Tris-HCl pH 7.0 containing 1.5% β−octyl-glucopiranoside (OG) and 1 M NaCl, overnight at 4 °C. The extract was centrifuged at 1000× *g* to avoid precipitates and extensively dialyzed against 20 mM Tris-HCl pH 7.0. The dialysate was re-centrifuged at 20,000× *g* in the Hitachi centrifuge to eliminate few small precipitates of unknown origin and filtered through a 0.2 μm pore size membrane (Minisart filter, Millipore, Sigma-Aldrich, Merck, Darmstadt, Germany).

### 2.7. Incubation of Xenopus Oocyte or MeT5a Membrane-Rich Fractions with Asbestos Fibers 

To the 19 ml of extracts (containing 0.25 mg protein/ml for *Xenopus* oocytes, and 0.48 mg protein/ml for MeT5a cells), another aliquot of proteinase inhibitor cocktail was added and 1.2 mL were incubated with 0.18 mg Croc, 0.18 mg Woll or 0.18 mg Chry, all previously saturated with 1% BSA, for 90 min at room temperature, in a final volume of 1.5 mL of TRIS-HCl 20 mM pH 7.0 containing 0.15 M NaCl (TRIS) and washed in the same buffer. After this incubation, the mixtures were centrifuged at 10,000 rpm for 10 min in an Eppendorf microcentrifuge, the supernatants were stored at −20 °C and the pellets washed once in TRIS. The pellets containing the fibers with adsorbed proteins, were then re-suspended in 0.2 mL of SDS1% containing 0.3M NaCl and boiled for 15 min. After centrifugation, the resulting supernatants were withdrawn carefully and stored at −20 °C and analyzed in Western blotting and revealed using 1:200 anti-TMEM16A rabbit antibodies (Abcam 53212, Cambridge, UK) as primary antibody and anti-rabbit peroxidase conjugate as a secondary antibody (Abcam 6721, Cambridge, UK). Western Blot images were processed using the free open-source raster graphics editor GIMP. The images were converted into grayscale (Image->Mode->Grayscale) and some changes in brightness and contrast were equally applied across the entire image. More details are shown in Supplemental Materials (Figure S1), where it is reported the scanning of the full film and the indicated cropped area used to prepare the final figure.

### 2.8. Statistical Analysis

Prism 4.0 (GraphPad Software, San Diego, CA, USA) was used for the statistical analysis. All data passed the normality test (Kolmogorov—Smirnov test). Statistical significance for comparison between two different groups was established using a Student’s t test or ANOVA for multiple comparisons (more details of the analysis are specified in Figure legends). All values are expressed as mean ± SEM. 

## 3. Results

### 3.1. The Croc-Sensitive Current Is Mediated by TMEM16A Channels Endogenously Expressed in the Oocyte Cell Membrane

**Figure 1:**
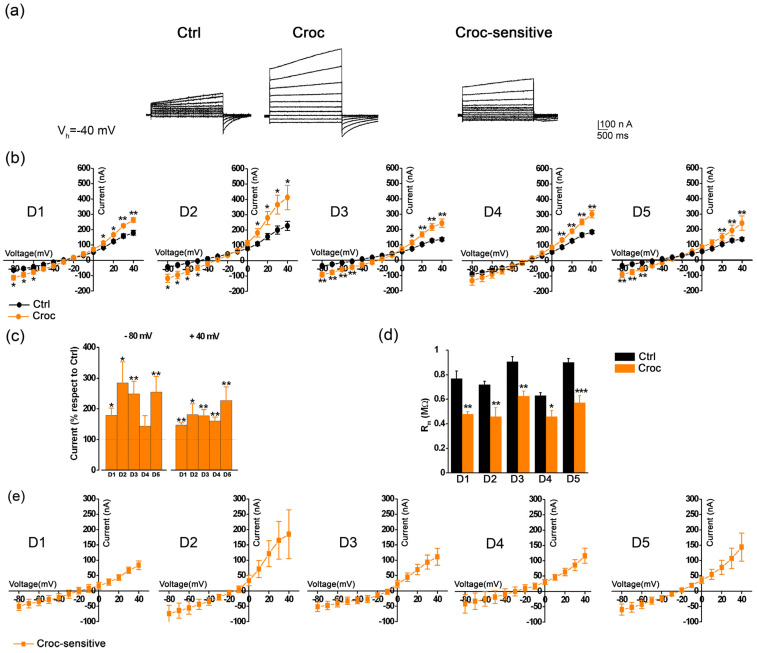
(**a**) Example of evoked currents recorded in untreated (Ctrl) and asbestos-treated (Croc) *Xenopus* oocyte cells, and the *Croc-sensitive currents* obtained by their subtraction; cells were held at −40 mV, then stepped from −80 mV to + 40 mV for 3 s. (**b**) The I–V relationships of Ctrl and Croc-treated cells derived from 5 frog donors (D1–D5). (**c**) Averages of the peak current amplitudes recorded at −80 mV and +40 mV measured as a percentage of their respective Ctrl (of the same batch). (**d**) Comparison of the R_m_ (membrane resistance) values, before and after the Croc-treatment (same cells of b). (**e**) I–V relationships obtained by plotting the averages of the *Croc-sensitive currents* derived from the same oocytes of (**b**). Note the strong outward rectification and the reversal of the currents around –20 mV under the present recording conditions. *n* ≥ 4 for each condition, * *p* < 0.05, ** *p* < 0.01, *** *p* < 0.001, unpaired *t*-test.

The hallmark properties of TMEM16A channels are their slow activation kinetics, strong outward rectification and a reversal potential near the Cl^−^ equilibrium potential. Figure 1a shows an example of the Croc effect on the current traces recorded by stepping the membrane of the *Xenopus* oocytes to different voltages under voltage clamp (V_h_ = −40 mV, from −80 mV to +40 mV); the treatment clearly increased the amplitude of the evoked outward current when compared to that of untreated (Ctrl) cells of the same batch, and visible from the *Croc-sensitive current* traces obtained from their subtraction that showed typical slow activation kinetics and a strong outward rectification. 

Despite the variability among oocytes from different donors, the effect of Croc was visible at both negative and positive membrane potentials, as observed from the averages of the I-V relationships obtained in different batches of cells isolated from 5 frog donors (D1–D5, Figure 1b), and from the percentage (with respect to Ctrl) of the current amplitude recorded at −80 mV and +40 mV of Figure 1c, showing Croc was able to significantly affect also the inward (leak) currents.

On average, Croc treatment reduced the membrane resistance (R_m_) from 0.82 ± 0.02 MΩ, *n* = 39 to 0.53 ± 0.03 ΜΩ (*n* = 33, *** *p* < 0.001, same cells of Figure 1b, unpaired *t*-test, data not shown), as also reported previously [3], and the tendency was consistent in all donors (Figure 1d). The I–V relationships of the *Croc-sensitive currents* derived from the subtraction of those in Figure 1b (Figure 1e), showed a mean reversal level of −18.84 ± 2.86 mV (*n* = 33, 5 donors, data not shown) near the equilibrium potential for Cl^−^, as reported in *Xenopus* oocytes recorded with KCl-filled microelectrodes [5].

**Figure 2:**
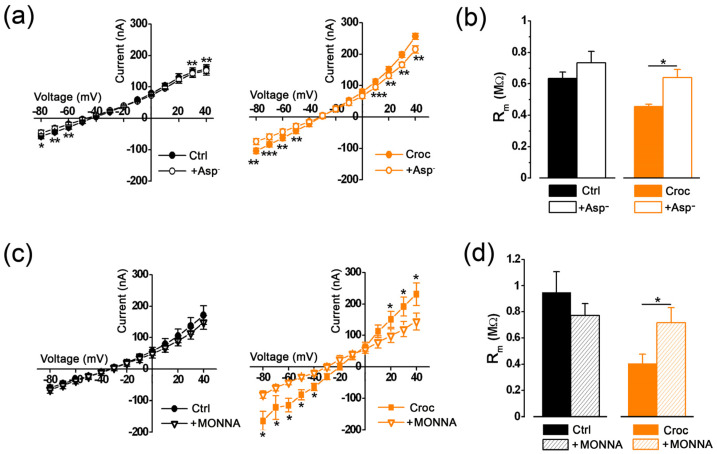
(**a**) I–V relationships of untreated (Ctrl, *n* = 4) and Croc-treated *Xenopus* oocyte cells (*n* = 5) before (left) and after (right) the partial replacement of [Cl^−^]_e_ with aspartate (cells of the same donor). (**b**) Effect of chloride replacement with aspartate on the membrane resistance (R_m_, Ctrl: 0.63 ± 0.004 MΩ, Ctrl + Asp: 0.73 ± 0.07 MΩ, *n* = 4; Croc: 0.46 ± 0.01 MΩ, Croc + Asp: 0.64 ± 0.05 MΩ, *n* = 5, same cells as in (**a**), paired *t*-test, * *p* < 0.05). (**c**) I–V relationships of untreated (Ctrl, *n* = 5) and Croc-treated cells (*n* = 5) in the absence (left) and in the presence (right) of MONNA (10 μM, same donor). Note that the effect was visible only on Croc-treated cells (* *p* < 0.05, paired *t*-test). (**d**) Effect of MONNA on the membrane resistance (R_m_, Ctrl: 0.95 ± 0.02 MΩ, Ctrl + MONNA: 0.77 ± 0.09 MΩ, *n* = 5; Croc: 0.41 ± 0.07 MΩ, Croc + MONNA: 0.71 ± 0.11 MΩ, *n* = 5, same cells as in (**c**), * *p* < 0.05, ** *p* < 0.01, *** *p* < 0.001, paired *t*-test).

To investigate the involvement of Cl^−^ in the *Croc-sensitive current*, half of the [Cl^−^]_e_ was replaced with an anion impermeable to the chloride channels, such as aspartate (Asp); as expected, the effect of Croc on the evoked currents was partially abolished, as inferred from the I-V relationships obtained before and after the [Cl^−^]_e_ replacement (Figure 2a right, ** *p* < 0.01, *** *p* < 0.001), as well as for those recorded in the Ctrl oocytes of the same batch (Figure 2a left, * *p* < 0.05, ** *p* < 0.01). The replacement also reduced the effect of Croc on R_m_, which now became similar to that of Ctrl cells (Figure 2b). In another batch of cells, a similar effect was observed in the presence of 10 μM MONNA ((N-((4-methoxy)-2-naphthyl)-5-nitroanthranilic acid), a specific blocker for the TMEM16A channels [18,23]; MONNA induced a substantial reduction in the evoked currents only in Croc-treated cells (Figure 2c) and significantly reversed the effect of Croc on R_m_ (from 0.41 ± 0.07 MΩ to 0.71 ± 0.11 MΩ, *n* = 5, * p < 0.05, Figure 2d). These findings strongly suggested that the TMEM16A channels were the mediator of the *Croc-sensitive current* in the *Xenopus* oocytes.

### 3.2. The Effect of Croc Exposure on the TMEM16A-Current Is Modulated by the [Ca^2+^]_e_

**Figure 3:**
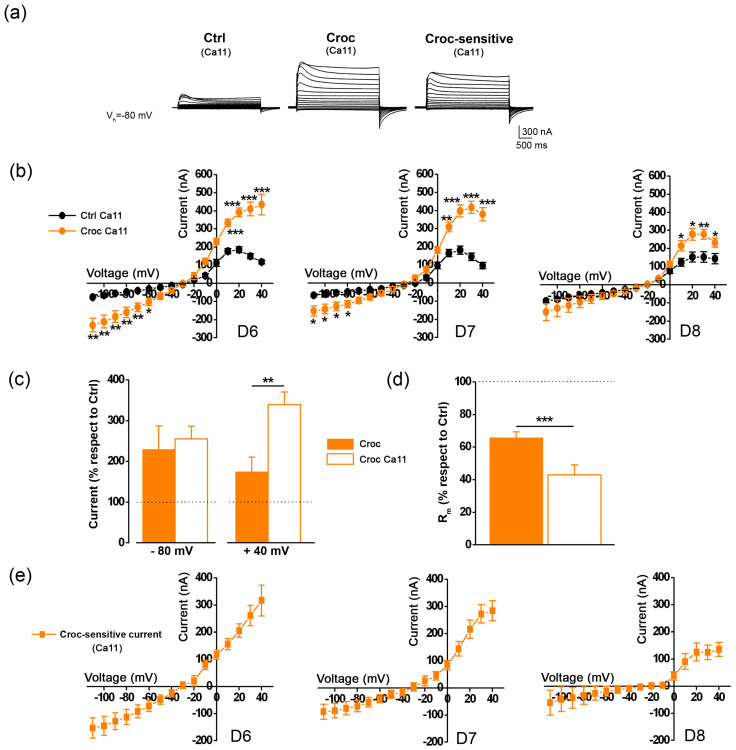
(**a**) Left, Example of currents recorded in the presence of [Ca^2+^]_e_ = 11 mM (Ca11) from a Ctrl and a Croc-treated cell. The traces were obtained by clamping the cell membrane at V_h_ = −80 mV, and then stepping from −110 mV to +40 mV (3-sec interval). Note the more prominent and changed kinetics of the transient outward currents in the high Ca^2+^ bathing solution recorded at positive potentials, which was still enhanced by Croc exposure. Right, the *Croc-sensitive currents* obtained by their subtraction. (**b**) The I–V relationships of the currents measured at the peak in cells derived from 3 frog donors (D6–8). (**c**) Percentage of current amplitude recorded in Croc-treated cells with respect to the Ctrl: note that in high Ca^2+^ bathing solution (Ca11), the percentage measured at +40 mV was higher than in normal Ringer. (**d**) Percentage of the R_m_ values recorded under this condition (Ca11) decreased with respect to that recorded in normal Ringer solution (same cells of Figure 1). (**e**) The averaged I–V relationships of the *Croc-sensitive currents* obtained from the subtraction of those of Figure 3a. * *p* < 0.05, ** *p* < 0.01, *** *p* < 0.001, unpaired *t*-test.

**Figure 4:**
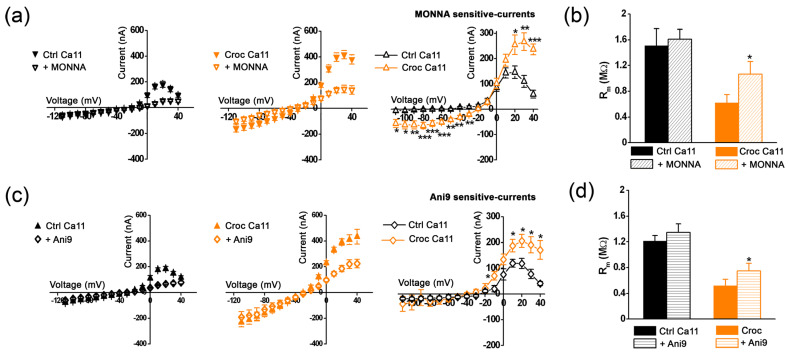
(**a**) The I–V relationships of the currents recorded in the presence of [Ca^2+^]_e_ =11 mM (Ca11) in the absence and in the presence of MONNA (10 μM). Right, The I–V relationships obtained from their subtraction (MONNA-sensitive currents, unpaired *t*-test). (**b**) The effect of MONNA on R_m_ (same cells of a, paired t-test, values in the text). (**c**) Left, the I–V relationships of the currents recorded in the presence of [Ca^2+^]_e_ =11 mM in Ctrl and Croc-treated cells, in the absence and in the presence of Ani9 (1 μM). Right, the I–V relationships obtained from their subtraction (unpaired *t*-test). (**d**) The effect of Ani9 on R_m_ (same cells of c, paired t-test, values in the text). * *p* < 0.05, ** *p* < 0.01, *** *p* < 0.001.

The next series of experiments were aimed at further characterizing the *Croc-sensitive currents* by adapting different protocols to test the Ca^2+^ and voltage sensitivity, and the pharmacology of the TMEM16A channels. By holding the membrane potential at −80 mV in high [Ca^2+^]_e_ (Ca11 = 11 mM), a transient outward current was clearly visible on stepping to a positive potential, in both untreated and Croc-treated cells (Figure 3a). The current began to appear at membrane potentials more positive than −20 mV, and it reached a peak around +20 mV, in line to what was previously reported in the literature for the endogenous Ca^2+^-dependent Cl^−^ currents recorded in oocytes under similar conditions (Figure 3b) [5]. However, in Ca11 the percentage of the current amplitude of Croc-treated cells (with respect to Ctrl cells) recorded at negative membrane potentials increased similarly to that in normal Ringer solution (e.g., −80 mV; Ca11: 255.97 ± 31% *n* = 20; Ringer: 229.97 ± 57%, *n* = 31), while at positive membrane potential, the percentage was significantly different; e.g., at + 40 mV in normal Ringer solution, the increase was 175 ± 51 % more than Ctrl (Figure 3c, *n* = 31, same donors as of Figure 1), in high Ca^2+^ the increase was further potentiated to 339 ± 31% with respect to the amplitude recorded in Ctrl cells under the same condition (Figure 3c, *n* = 20, same donors of Figure 3b, *** *p* < 0.001). Despite the donor’s variability, the R_m_ tended to decrease following Croc-exposure, even more than that in normal Ringer solution (Figure 3d, left, Ringer: 65.84 ± 3.46% of the Ctrl, *n* = 33 same cells of Figure 1; Ca11: 43.31 ± 5.78% of the Ctrl, *n* = 20, *** *p* < 0.001). The *Croc-sensitive currents* recorded in high Ca^2+^ (Figure 4e) also reversed near the equilibrium potential of chloride (−28.64 ± 6.96 mV, *n* = 20, data not shown), although this was slightly more negative than that recorded in Ringer (*p* = 0.14, unpaired *t*-test).

Next, we compared the effect MONNA (10 μM) with Ani9 (2-(4-chloro-2-methylphenoxy)-*N*′-(2-methoxybenzylidene) acetohydrazide), another blocker for TMEM16A channels [19]. The I–V relationships of the currents recorded in high [Ca^2+^]_e_ in Ctrl and Croc-treated oocytes and MONNA-sensitive currents obtained from their subtractions are shown in Figure 4a. The MONNA treatment partially recovered the effect of Croc on R_m_ (Croc: 0.62 ± 0.23 MΩ; Croc + MONNA: 1.07 ± 0.19 MΩ; *n* = 9, * *p* < 0.05, Figure 4b). The application of Ani9 (1 μM) also reduced the evoked outward current; similar to MONNA, the amplitude of the Ani9-sensitive currents was potentiated by the Croc-exposure (Figure 4c). In line with the effect of MONNA, Ani9 partially recovered the reduction of the R_m_ values following asbestos treatment (Croc: 0.52 ± 0.10 MΩ; Croc + Ani9: 0.75 ± 0.12 MΩ; *n* = 8, * *p* < 0.05, Figure 4d).

**Figure 5:**
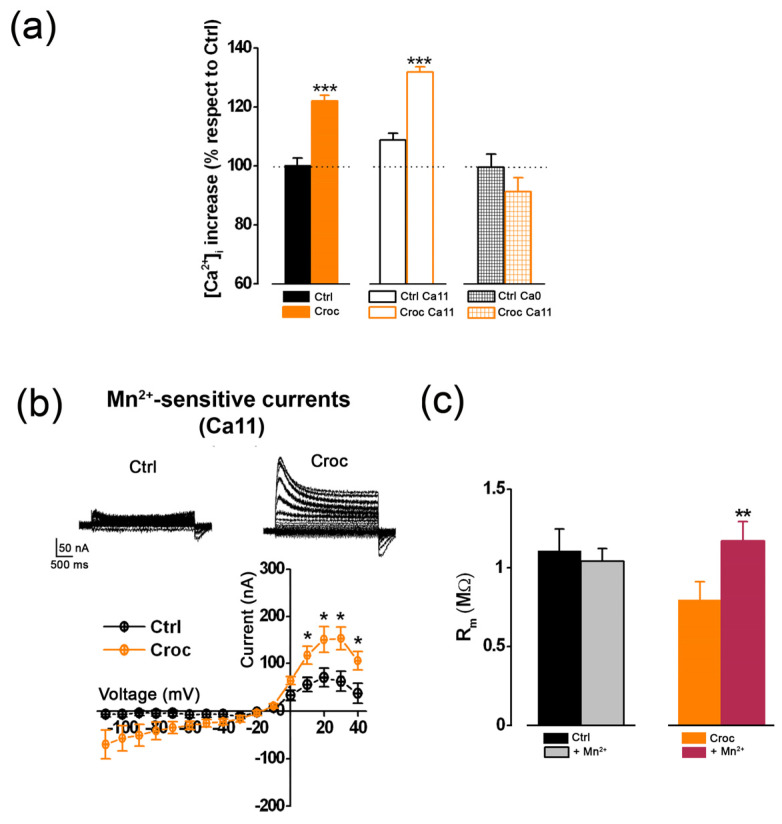
(**a**) Resting [Ca^2+^]_i_ increased in normal Ringer’s solution (Ctrl: *n* = 17; Croc: *n* = 9), in high [Ca^2+^]_e_ ([Ca11]_e_ = 11 mM, Ctrl Ca11: *n* = 17, Croc Ca11: *n* = 14) and lack of increase in Ca^2+^-free solution (Ca0, Ctrl Ca0: *n* = 13, Croc Ca11: *n* = 10) measured as a percentage relative to Ctrl oocytes (in normal Ringer solution) from the same frog donor (2 donors, ANOVA Dunnett’s test). (**b**) Example of recording traces (subtraction currents-see Figure 1a and Figure 3a) showing blocking effect of Mn^2+^ (5 mM) in Croc-treated oocyte, compared with Ctrl and the I–V relationships of the Mn^2+^-sensitive currents obtained from *n* = 7 (Ctrl) and *n* = 6 (Croc) oocytes (Ca11, unpaired t-test, same batch). (**c**) The application of Mn^2+^ also restored the normal effect of Croc on R_m_ (decrease) back to control level (paired *t*-test, same cells of c). * *p* < 0.05, ** *p* < 0.01, *** *p* < 0.001.

These results clearly suggested that in Ca11, the *Croc-sensitive chloride current* as well as the effect of Croc on R_m_, were enhanced, most likely by an increased Ca^2+^ influx across the cell membrane. To further investigate such a hypothesis, a set of [Ca^2+^]_i_ measurements was conducted by using a Ca^2+^ imaging approach. Briefly, oocytes were microinjected with Fura 2 and the [Ca^2+^]_i_ levels were measured as mean 340/380 fluorescence values (see Materials and Methods for further details). In accordance with what has previously been reported in the literature, preparatory experiments revealed that oocyte incubation in Ca11 did not significantly alter the [Ca^2+^]_i_ with respect to oocytes bathed in normal Ringer (Ca11 vs. Ringer: 8.71 ± 2.23% increment, *n* = 17, *p* > 0.05; Figure 5a), confirming that the oocyte cell membrane at rest is poorly permeable to this cation [24]. On the other hand, in oocytes exposed to Croc in normal Ringer solution, the [Ca^2+^]_i_ was 21.99 ± 1.99% higher than Ctrl (Ctrl: *n* = 17; Croc: *n* = 9, *** *p* < 0.001) and the percentage increased to 31.83 ± 1.77 % when the oocyte exposure was carried out in Ca11 (*n* = 14, *** *p* < 0.001 vs. Ctrl; Figure 5a). This observation suggested that the Croc exposure significantly increased the resting membrane permeability to Ca^2+^. Accordingly, a reduction of [Ca^2+^]_i_ was observed when the Croc exposure was conducted in oocytes bathed in a Ca^2+^-free solution which became similar to Ctrl cells under the same condition (Ctrl Ca0: 99.51± 4.45%, *n* = 13; Croc Ca0: 91.22 ± 4.83% *n* = 10, *p* > 0.05, Figure 5a). Control experiments showed that the incubation of the oocytes in Ca0 did not alter the [Ca^2+^]_i_ per se, excluding any side effect due to the absence of Ca^2+^ in the extracellular solution (Ctrl: 100.31± 2.31% *n* = 17; Ctrl Ca0: 99.51 ± 4.45%, *n* = 13, *p* > 0.05).

The Ca^2+^ membrane permeability of the oocyte is usually very low, especially at rest, and mainly carried by voltage-gated calcium channels (VOCC) [25,26]. Surprisingly, the pharmacology of these channels is still poorly described, although they are blocked by divalent cations such as Mn^2+^ (5 mM) [5,25]. Similar to TMEM16A blockers, Mn^2+^ (5 mM) also reduced the chloride currents induced by stepping the voltage of the cell membrane in Croc-treated cells (V_h_ = −80 mV, Figure 5b). However, Mn^2+^ could also be blocking some other sources of Ca^2+^ entry, different than VOCC [27]. In line with this idea, the cation fully recovered the effect of asbestos on R_m_ (Figure 5c) suggesting that Mn^2+^ was able to reduce the Ca^2+^-membrane permeability and the tonic activation of the TMEM16A channels at rest.

### 3.3. Western Blotting Experiments

**Figure 6:**
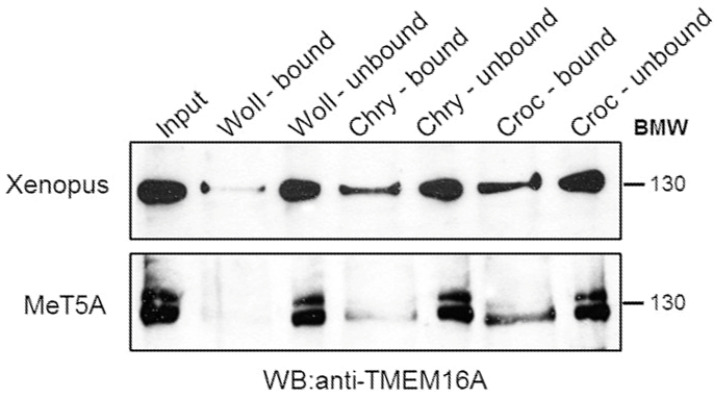
Western blotting showing that TMEM16A was bound by fibers either in oocytes or MeT5a membrane-rich fractions and spun down with them. From left to right: lane 1 (upper and lower) total amount of membrane-rich fractions, 30 μg protein loaded; lane 2 (upper and lower) 50 μL of Woll pellets (resuspended in 200 μL) incubated with either oocytes or MeT5A membrane-rich fractions; lane 3, (upper and lower) 50 μL of supernatant of mixture Woll-membrane-rich fractions; lane 4, (upper and lower) 50 μL of Chry pellets (resuspended in 200 μL) incubated with either oocytes or MeT5A membrane-rich fractions; lane 5 (upper and lower) 50 μL of supernatant of mixture Chry-membrane-rich fractions; lane 6, (upper and lower) 50 μL of Croc pellets (resuspended in 200 μL) incubated with either oocytes or MeT5A membrane-rich fractions; lane 7, (upper and lower) 50 μL of the supernatant of the mixture Croc-membrane-rich fractions. It is worth noting that the main signal was obtained with Croc in either case.

Western blot experiments showed that the TMEM16A antibody reacts with *Xenopus* as well as human TMEM16A expressed in the human mesothelial cell line (MeT5a) suggesting a very conserved structure for this protein. Following interaction of asbestos fibers with *Xenopus* oocyte membrane extracts, Croc adsorbed consistently more to TMEM16A than Chry (chrysotile), while Woll (wollastonite) showed a very weak signal. Interestingly, similar results were obtained following the incubation of asbestos fibers with a membrane-rich extract of MeT5a cells (Figure 6). Two bands corresponding to TMEM16A were observed in the human cell line. In human cells, TMEM16A is alternatively spliced, generating four isoforms [28]; however, the small difference in molecular weight between the two detected bands in our experimental conditions suggested the presence of a glycosylated form rather than a spliced one.

## 4. Discussion

In addition to the effect of asbestos fiber exposure on the passive membrane properties (R_m_ and RP) and the increase in the outward current amplitude induced by depolarizing steps already described in our previous studies [3,4], here we identify TMEM16A channels as a new molecular player in the biological effect of asbestos fibers in *Xenopus* oocytes. Our conclusion arose from data showing that Croc exposure caused an alteration of the Cl^−^ membrane permeability and a strong reduction in the effect of the Croc treatment after replacing external Cl^−^ ion with aspartate. Moreover, our experiments revealed a potentiation of the effect of the Croc treatment by increasing the [Ca^2+^]_e_ from 1.8 to 11 mM and by stepping the voltages of the membrane from a more hyperpolarized level V_h_ (−80 mV) and the blockage of the *Croc-sensitive currents* by the specific TMEM16A channel antagonists MONNA and Ani9.

Concerning the role of TMEM16A in mediating the effect of the asbestos fiber exposure on the passive oocyte membrane properties, here we observed that the reduction in R_m_ caused by the Croc-treatment was partially restored by the specific TMEM16A antagonists suggesting a “tonic” activation of the TMEM16A channels following the Croc-treatment. Accordingly, the overexpression of TMEM16A channels has already been demonstrated to lead to a reduction in the R_m_ in *Xenopus* oocytes [8].

At least two types of CaCCs currents have been described in *Xenopus* oocytes. The first is induced by a “standard” protocol (stepping positive from V_h_ = −40 mV in normal Ringer) [6] and a second (transient) outward current elicited from a more hyperpolarized V_h_ (−80 mV or −100 mV) as well by increasing the [Ca^2+^]_e_ [5,29]. Both currents were observed in our experimental conditions (Figure 1a and Figure 3a). *Xenopus* oocytes have long been considered a convenient model for studying CaCCs, in view of these channels being the predominant type natively expressed in these cells. The expression of *Xenopus* TMEM16A in *Axolotl* oocytes produces evoked outward currents with multiple components [6], supporting the idea that CaCC currents recorded in *Xenopus* oocytes are also likely to be carried by the same type of channels [23]. 

At least two “sources” could be the responsible for any variation of the [Ca^2+^]_i_ following step depolarization: Ca^2+^ released from internal stores (in oocytes, mostly the IP_3_-sensitive stores) or the Ca^2+^ influx from the extracellular space. Here, we found that in Ca^2+^-free Ringer solution, the [Ca^2+^]_i_ of Croc-treated cells was similar to Ctrl cells. On the other hand, in normal condition, the Croc exposure increased the [Ca^2+^]_i_ with respect to non-treated cells. This finding supports the Ca^2+^ influx from the extracellular space as a predominant source of Ca^2+^ induced by the asbestos fibers.

Additionally, considering our previous studies [3,4], we suggest at least three possible mechanisms by which Croc could increase the Ca^2+^ influx and thus the *“resting”* [Ca^2+^]_i_ (as we observed flourometrically) and TMEM16A activation in *Xenopus* oocytes; 1) by increasing the Ca^2+^ influx through VOCC; 2) by forming membrane “pores” permeable to Ca^2+^ [3]; 3) or by modulating Ca^2+^ currents from some unknown leak channels [27,30]. All of them could increase the [Ca^2+^]_i_ and thus induce a tonic activation of the TMEM16A channel activity, altering in this way the passive membrane properties of the cells. One more possibility must be also mentioned. Many proteins, with different structures, molecular weight and isoelectric point are adsorbed with a specific profile by specific asbestos fibers, suggesting a specific interaction [31]. Several authors have investigated the extent of adsorbed proteins and the nature of these, but only rarely have analyzed the function of the adsorbed protein. Borelli et al. (2007) [32] and Pascolo et al. (2015) [33], showed that the so-called asbestos “body” consists of the asbestos fiber covered by ferritin which was shown to become unfolded, and Borelli et al. (2018) [34] reported that mast cell enzymes undergo a significant activation following asbestos binding. These findings suggest that after asbestos fiber adsorption, the target protein can be modified both in its structure and function. Our Western blotting analysis showed that Croc binds TMEM16A proteins more than the other fibers tested. Therefore, we could speculate an enhancing channel activity effect mediated by a direct interaction of Croc with the TMEM16A protein. In support of this, Woll who just poorly pulled-down the channel, did not elicit any electrical effect on *Xenopus* cell membranes [3] nor has it been considered a carcinogen [35] suggesting a cause-and-effect relationship between electrical changes and carcinogenesis. Chry also bound TMEM16A, even though to a much lesser extent as compared to Croc, and its effect on the channel activity is currently under investigation. 

Interestingly, the asbestos fiber absorption to TMEM16A was detected also by incubating an extract of a membrane-rich fraction obtained from a human mesothelial cell line (MeT5a). While oocytes express only one isoform of TMEM16A, two isoforms were expressed by MeT5a cells and the asbestos fibers mainly absorbed the lower isoform. Explaining why one human isoform shows a higher affinity for Croc fibers was beyond the aim of the present study; however, the results indicated a possible direct TMEM16A-fiber interaction in *Xenopus* oocytes as well as in human cells. If this is the case, this interaction could also modulate some TMEM16A channel properties, such as the Ca^2+^-and/or voltage sensitivity. This aspect deserves to be investigated in the future. 

**Figure 7:**
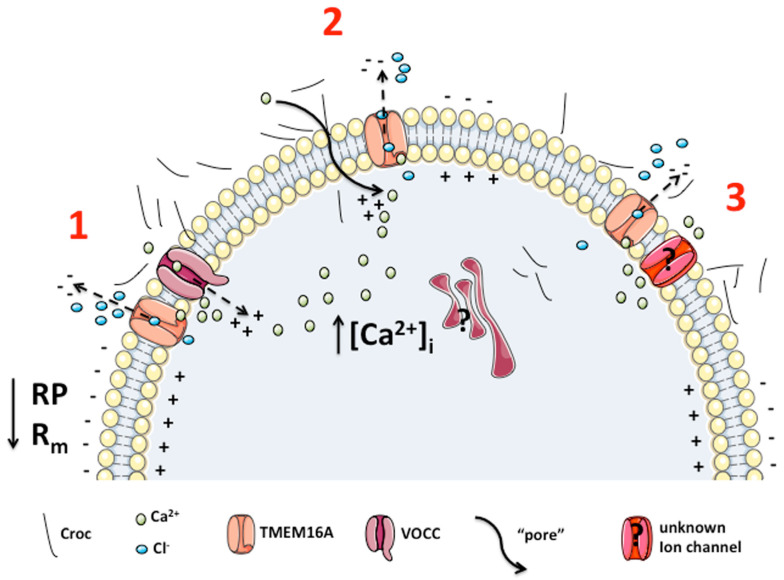
Schematic representation of the possible mechanisms involved in Croc-induced TMEM16A activation in *Xenopus* oocytes. We propose the increment in [Ca^2+^]_i_ required for TMEM16A activation is derived mainly from the extracellular environment. The possible responsible mechanisms are: (1) potentiation of VOCC activity (e.g., voltage operated calcium channels); (2) membrane lesions/perturbations induced directly by the penetration of Croc fibers [3]; (3) undefined ‘leak’ ion channels permeable to Ca^2+^ [27,30]. At point 2, the possibility of a direct absorption of TMEM16A with asbestos fibers is also illustrated. All the above Croc-mediated effects on TMEM16A channel activity could explain the effects on the passive oocyte membrane properties, such as the membrane resistance (R_m_) and the resting membrane potential (RP, previously reported in Bernareggi et al., 2015, 2019 [3,4]). However, at the moment, we cannot exclude other additional pathways such those involving internal structures, as well as any direct interaction of the fibers with the TMEM16A protein itself (not shown, discussed in the text). Both deserve to be investigated in future studies. The Figure was partly generated using Servier Medical Art, provided by Servier, licensed under a Creative Commons Attribution 3.0 unported license.

The possible scenario of the putative mechanisms through which asbestos fibers could affect TMEM16A channel activity, on the basis of our findings and previous literature is depicted in Figure 7, including any possible intracellular triggering mechanisms not yet investigated. Further investigations are required to dissect all the possibilities.

The evidence for TMEM16A gene amplification and over expression in several types of tumors is well consolidated [9,13,14,15,16,17]. The relationship between TMEM16A expression/activity and tumor growth/invasion has been demonstrated in human prostate carcinoma cells both in vivo and in vitro [36,37]. The hypothesis of the TMEM16A channel as an indirect or even direct membrane molecular player in the asbestos-associated pathologies in humans has been proposed by a wide range of experimental work and stressed in excellent reviews. In addition, also our bioinformatic analysis (see Supplementary Figures S2 and S3, [38,39,40,41,42]) suggests that this molecule can play a key role in the development of asbestos-related tumors as well (i.e., head and neck squamous cell carcinoma, lung adenocarcinoma, lung squamous cell carcinoma) and that the overexpression of TMEM16A channels could represent a good prognostic factor.

Although the mechanism through which TMEM16A exerts the tumorigenic activity is not completely clear, recent studies suggest that it interacts with the Ezrin-Radixin-Moesin (ERM) network in human embryonic kidney (HEK) cells [43]. Considering that the ERM network is an intermediate in the connection between the actin cytoskeleton and the plasma membrane, the TMEM16A-ERM network may regulate cancer cell migration, invasion or adhesion [44]. Interestingly, the overexpression of TMEM16A was also reported to affect the morphology of the cell membrane, via the ERM network in *Xenopus* oocytes [36] and we have previously shown that *Xenopus* oocyte membranes undergo morphological changes following asbestos fiber exposure [3]. 

## 5. Conclusions

Taking advantage of the *Xenopus* oocyte model, the present study strongly implicates TMEM16A channels as key molecular participants in the Croc asbestos-mediated effects on cell membranes. The alteration of Ca^2+^ homeostasis is a well-known tumorigenic hallmark in lung cancer [45]. Our findings suggest a cross-talk between the [Ca^2+^]_i_ variation observed during asbestos exposure and the activation of TMEM16A channels. Asbestos fibers can also affect cell function both directly via ROS production, induction of membrane lesions [34] and following induction of iron overload, induction of ferroptosis and mutagenicity [46,47]. Activation of TMEM16A can strongly contribute to the tumor promoting activity of asbestos by enhancing cell motility and growth. Consequently, TMEM16A channels may have a role in tumorigenesis in the pathophysiology of asbestos-related human tumors. In this perspective, we think that the use of TMEM16A channels as a new therapeutic target and/or prognostic marker in the management of asbestos-related diseases would merit future attention. Each type of asbestos may be involved in tumorigenesis. However, the role of Chry in this sense is controversial with respect to that of Croc [48]. Therefore, a relationship between electrophysiological changes induced in our model and tumorigenesis is presently difficult to draw. The differential contribution of these fibers in affecting the electrophysiological properties of *Xenopus* oocytes will be addressed in a future dedicated research program.

## Data Availability

Not applicable.

## Supplementary Materials

The following supporting information can be downloaded at: https://www.mdpi.com/article/10.3390/membranes13020180/s1.
