# Supplementary material for: Asbestos Fibers Enhance the TMEM16A Channel Activity in Xenopus Oocytes
Source: Membranes (Basel). 2023 Feb 1;13(2):180. doi: 10.3390/membranes13020180 (PMC9960392; doi:10.3390/membranes13020180)
Supplement: Supplementary file 1 [file membranes-13-00180-s001.zip › membranes-2167129-supplementary.pdf]

Supplementary Information

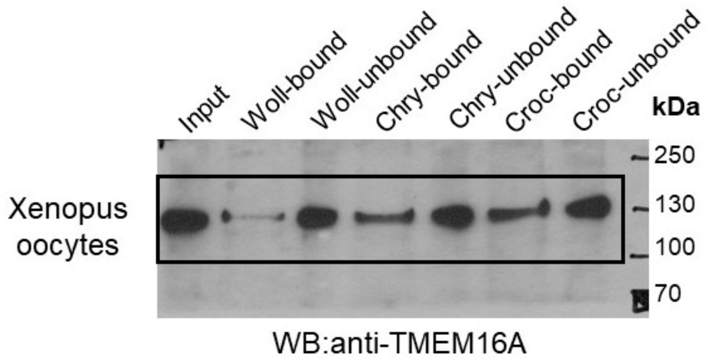

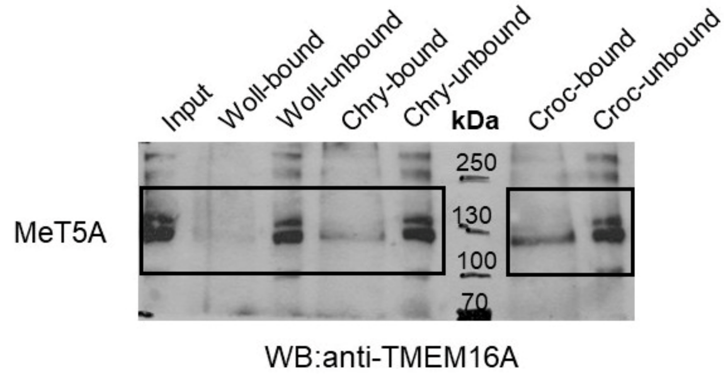

Figure S1. Western blots showing TMEM16A expression in *Xenopus* oocytes and MeT5A cells. Scanning of the full film and cropped areas were used to prepare Figure 6.

Bioinformatic analysis of TMEM16A expression in asbestos-related tumors in humans.

In order to provide more insight into the "*in vivo*" role of the TMEM16A Cl^- channel (also known as ANO1), and make its correlation stronger with asbestos exposure, we carried out a bioinformatic analysis on the mRNA expression in three asbestos-related human tumors: [38], lung adenocarcinoma (LUAD) [39], lung squamous cell carcinoma (LUSC) [39], and head and neck squamous cell carcinoma (HNSC) [40] using the UALCAN integrated data mining tool (http://ualcan.path.uab.edu/index.html) from "The Cancer Genome Atlas" [41]. The prognostic significance of TMEM16A expression and survival in LUAD, LUSC and HNSC was analyzed by the UALCAN tool [41]. Figure S1 shows that on average in LUSC samples, the mRNA for TMEM16A was reduced significantly (P < 0.001) with respect to control cell samples. The opposite was true for HNSC. In this case, the TMEM16A mRNA was *overexpressed* with respect to control cells (P < 0.001), while in LUAD no significant difference was found on the average. In Figure S2, the bioinformatic analysis revealed that LUAD patients with tumor tissue samples highly expressing TMEM16A, showed an overall survival that was significantly reduced with respect to those showing low expression (p=0.013). The same trend was found when HNSC samples were considered (p=0.00016). Therefore, TEME16A is a relevant prognostic factor and should be considered as one of the asbestos targets coming in contact firstly with fibers and possibly determining the subsequent cellular events leading to neoplastic transformation.

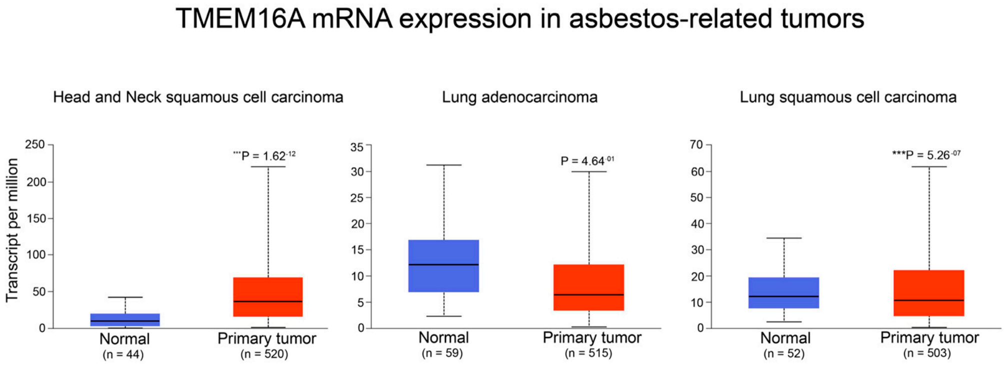

Figure S2. TMEM16A mRNA expression in asbestos related-tumors in human beings. By using the UALCAN data-mining platform from "The Cancer Genome Atlas" (http://ualcan.path.uab.edu/index.html) bioinformatics analysis was carried out on the TMEM16A mRNA expression in three asbestos-related tumors, *i.e.* head and neck squamous cell carcinoma, lung adenocarcinoma, and lung squamous cell carcinoma. The expression was highly heterogenous. The difference in TMEM16A gene expression between three cancers and normal tissue was analyzed by t-test. Note that it was increased for the neck squamous cell carcinoma patients and decreased for lung squamous cell carcinoma patients. Data are indicated as mean ± SD. The data used were analyzed by online statistical analysis. P values < 0.05 were considered as significant.

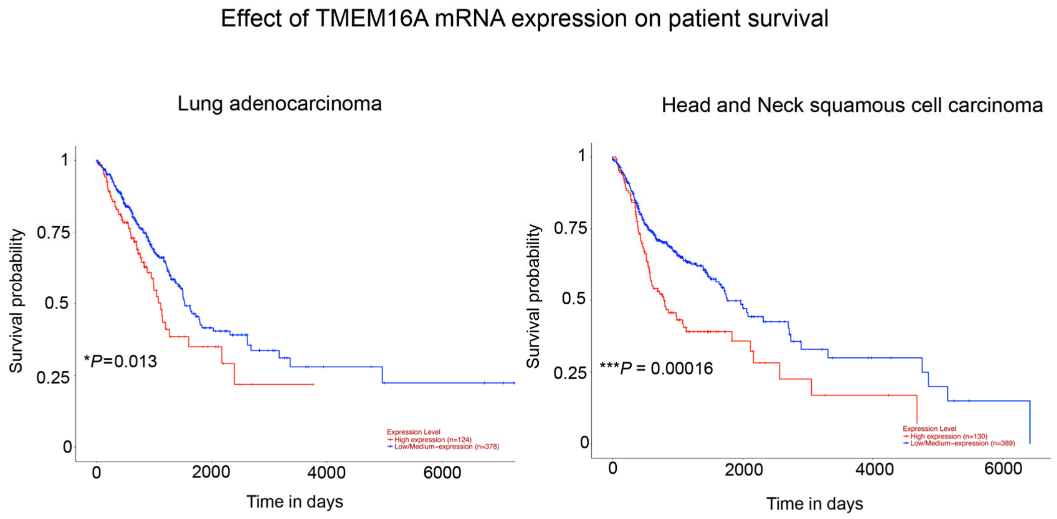

Figure S3. Overall survival of patients showing high expression of the gene for TMEM16A *vs* those showing a low expression. Comparison of the survival trends (low *vs.* high mRNA expression) for the same LUAD and HNSC patients of Figure S2. In agreement with literature [48], and with HNSC patients, LUAD patients that displayed tumors with a high expression of TMEM16A showed also an overall survival (OS) significantly reduced with respect to those showing a low expression. The relationship between TMEM16A gene expression and prognosis was analyzed using the Kaplan-Meier method. The Log-Rank test was used to assess the survival difference between patient groups. The data used were analyzed by online statistical analysis. P values <0.05 were considered as significant.
